# New insights into the surface plasmon resonance (SPR) driven photocatalytic H_2_ production of Au–TiO_2_†

**DOI:** 10.1039/c8ra05450a

**Published:** 2018-07-19

**Authors:** Jinlin Nie, Jenny Schneider, Fabian Sieland, Long Zhou, Shuwei Xia, Detlef W. Bahnemann

**Affiliations:** Key Laboratory of Marine Chemistry Theory and Technology, Ministry of Education, College of Chemistry and Chemical Engineering, Ocean University of China Songling Road 238 266100 Qingdao China shuweixia@ouc.edu.cn; Institut für Technische Chemie, Leibniz Universität Hannover Callinstr. 3 D-30167 Hannover Germany bahnemann@iftc.uni-hannover.de; Laboratory “Photoactive Nanocomposite Materials”, Saint-Petersburg State University Ulyanovskaya Str. 1, Peterhof 198504 Saint-Petersburg Russia

## Abstract

The Surface Plasmon Resonance (SPR) driven photocatalytic H_2_ production upon visible light illumination (≥500 nm) was investigated on gold-loaded TiO_2_ (Au–TiO_2_). It has been clearly shown that the Au-SPR can directly lead to photocatalytic H_2_ evolution under illumination (≥500 nm). However, there are still some open issues about the underlying mechanism for the SPR-driven photocatalytic H_2_ production, especially the explanation of the resonance energy transfer (RET) theory and the direct electron transfer (DET) theory. In this contribution, by means of the EPR and laser flash photolysis spectroscopy, we clearly showed the signals for different species formed by trapped electrons and holes in TiO_2_ upon visible light illumination (≥500 nm). However, the energy of the Au-SPR is insufficient to overcome the bandgap of TiO_2_. The signals of the trapped electrons and holes originate from two distinct processes, rather than the simple electron–hole pair excitation. Results obtained by Laser Flash Photolysis spectroscopy evidenced that, due to the Au-SPR effect, Au NPs can inject electrons to the conduction band of TiO_2_ and the Au-SPR can also initiate e^−^/h^+^ pair generation (interfacial charge transfer process) upon visible light illumination (≥500 nm). Moreover, the Density Functional Theory (DFT) calculation provided direct evidence that, due to the Au-SPR, new impurity energy levels occurred, thus further theoretically elaborating the proposed mechanisms.

## Abstract 2

The Surface Plasmon Resonance (SPR) driven photocatalytic H_2_ production upon visible light illumination (≥500 nm) was investigated on gold-loaded TiO_2_ (Au–TiO_2_).

## Graphical abstract

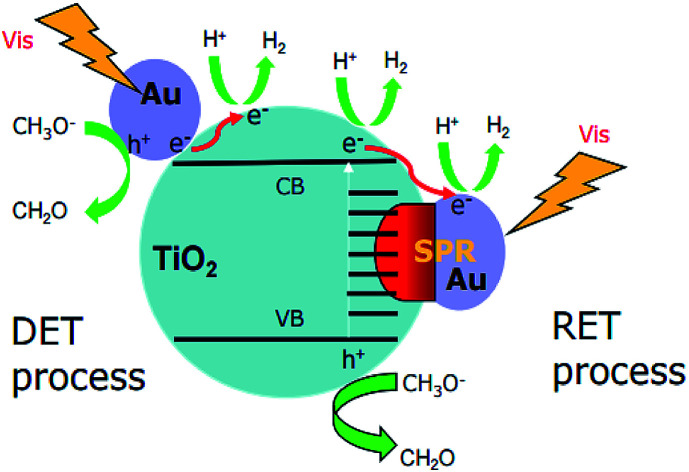

## 1. Introduction

Photocatalytic H_2_ evolution from water utilizing sun light is gaining importance for providing clean and sustainable energy.^1–3^ However, for practical applications, photocatalysts are desired to work efficiently under visible light which accounts for nearly half of the solar light.^4^ Recently, the rapid advances in plasmonic photocatalysis have accelerated the progress in the visible-light harvesting ability of TiO_2_-based photocatalysts. For instance, it has been reported that the surface-loading of Au nanoparticles can greatly increase the photocatalytic H_2_ production ability of Au–TiO_2_ upon visible light illumination.^4–9^

**Fig. 1:**
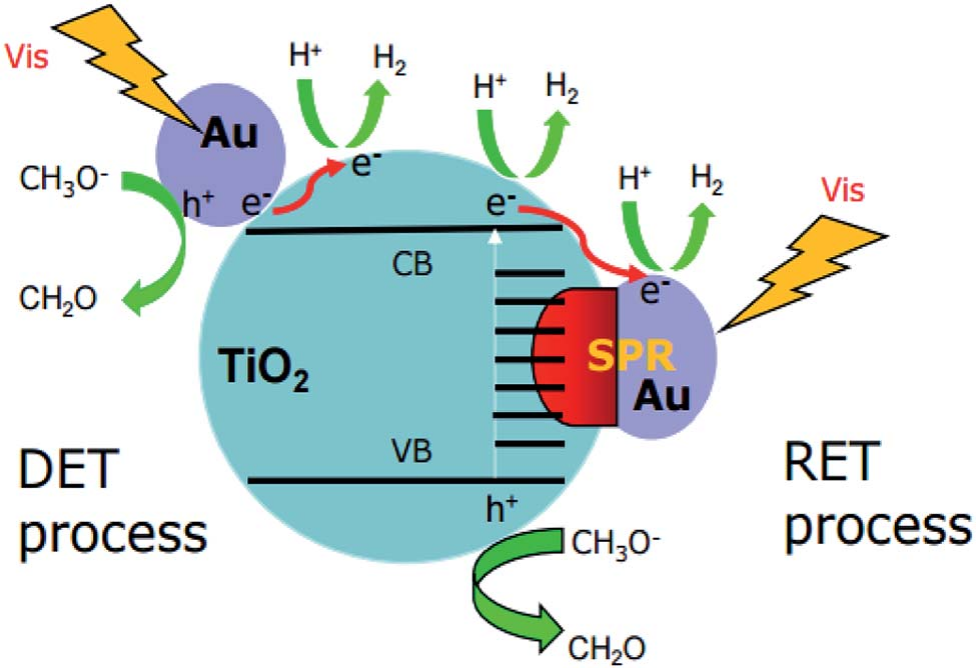
Two main proposed mechanisms for H_2_ production using Au–TiO_2_ in water/methanol mixtures under visible light illumination: direct electron transfer (DET) process (left); resonance energy transfer (RET) process (right).

However, the underlying mechanism remains unclear in the respective literatures.^4*a*,10–14^ Generally, two main mechanisms have been proposed to explain the visible light activity of Au–TiO_2_ photocatalysts (summarized in Fig. 1). The first mechanism known as resonance energy transfer (RET) is based on the idea that the SPR enhances the local electromagnetic field which in turn facilitates the generation of e^−^/h^+^ pairs near the semiconductor surface;^3,11,12^ D. B. Ingram^11^ and Z. W. Seh^12^ proposed this mechanism according to their results based on finite-difference time-domain (FDTD) simulations and discrete-dipole approximation (DDA) simulations, respectively, yet without exhibiting any experimental evidence. In addition, K. Awazu^15^ also obtained results supporting this mechanism when adding an insulating layer between the noble metal nanoparticles and the semiconductor. In this study, the plasmonic metal nanoparticles were homogeneously covered with a SiO_2_ shell thus preventing the direct electron transfer process. However, due to the energy transfer the enhanced photocatalytic activity was still achieved. The second mechanism explains the visible activity of Au–TiO_2_ by the SPR-excited electron transfer from the Au nanoparticles to the conduction band of TiO_2_, known as direct electron transfer (DET).^4*a*,10,13,16^ C. Gomes Silva^10^ and coworkers proposed this mechanism based on the observation that Au–TiO_2_ photocatalysts exhibit photocatalytic activity for H_2_ evolution upon 532 nm laser illumination. Recently, J. B. Priebe *et al.*^4*a*^ provided experimental evidence for the DET mechanism based on results obtained employing electron paramagnetic resonance (EPR) spectroscopy. In the EPR measurements, the authors detected the signals for electrons trapped at oxygen vacancies and for Ti^3+^ ions, which was considered as evidence for the electron transfer process from Au to TiO_2_. Herein, it was emphasized that the absorption of TiO_2_ is situated absolutely below 420 nm, therefore, the signals occurred due to the action of the Au nanoparticles rather than of TiO_2_. Moreover, it was excluded that the signals originated from electrons located in the Au nanoparticles themselves, as much higher *g* values and line widths are the requisites for these electrons to yield such signals.^4^ In conclusion, J. B. Priebe and co-worker argued that the obtained EPR-signals provide evidence for trapped electrons in TiO_2_ thus indicating a hot electron injection from the Au nanoparticles to TiO_2_. However, it should be mentioned here that the results obtained by these EPR measurements can also be explained by means of the RET mechanism, since according to this mechanism the SPR-enhanced electromagnetic field can initiate electron–hole pair excitation close to the TiO_2_ surface and thus the formation of the trapped electrons at this surface. Besides, due to pre-existing defects in TiO_2_,^11*b*,16^ such as oxygen vacancies, TiO_2_ exhibits also a small absorption shoulder above 400 nm indicative for surface defects. Actually, by means of EPR and laser flash photolysis spectroscopy, the obtained results clearly showed that bare TiO_2_ can be activated by the visible light illumination (≥420 nm) or using the 420 nm laser beam, which has long been neglected in the previous studies.^4*a*,11,12,17^

Therefore, the origin of the photoinduced electrons and the underlying mechanism upon visible light illumination remains unclear. Herein, we directly focus on the Au plasmon band, that is employing a 500 nm cutoff filter (≥500 nm) or using the 532 nm laser beam to totally get rid of the confusion caused by the direct excitation of TiO_2_.

## 2. Experimental section

### Photocatalyst preparation

Au–TiO_2_ photocatalysts consisting of 1 wt% gold on TiO_2_ (Evonik Aeroxide P25) were prepared by the typical sol immobilization (labeled as SIM) method,^4^ which was conducted by adding an aqueous solution of poly vinyl alcohol (PVA) (1.2 mL, 1 wt% sol, Aldrich, >99%) to 1 mL HAuCl_4_·3H_2_O (0.05 M). A freshly prepared NaBH_4_ solution (2.5 mL, 0.1 M, Aldrich, >96%) was added dropwise to the above solution, with the color turning dark. After 30 min, the TiO_2_ support (1.0 g) was added and the suspension was further stirred for 12 h at 25 °C. Then, the suspension was centrifuged, washed three times with distilled water and dried for 12 h at 70 °C. The as-prepared samples were used for catalytic and spectroscopic experiments without any other treatment.

### Material characterization

The UV-Vis absorption spectra were recorded employing a UV-visible spectrophotometer (Varian Cary 100 Bio). TEM images were characterized by Transmission Electron Microscopy (TEM) (FEI Tecnai G2 F20 TMP) using a 200 kV field emission gun (FEG). XRD patterns were monitored using a Bruker D8 Advance X-ray Diffraction system.

### Photocatalytic H_2_ evolution test

The photocatalytic hydrogen production experiments were conducted under argon atmosphere in a double-wall quartz glass reactor, with a cooling system (Julabo) maintaining the temperature at 20 °C. The 1 g L^−1^ photocatalyst suspensions were prepared by suspending Au (1 wt%)–TiO_2_ in methanol/water mixtures (volume ratio 1 : 5). The suspension was sonicated for 30 min before being transferred into the reactor and flushed with argon for 15 min to remove gases dissolved in the suspension and to make sure that Ar remained in the headspace of the reactor. The argon gas rate was kept constant at 10 cm^3^ min^−1^ during the photocatalytic measurement and the inlet gas flow rate for QMS was 1 cm^3^ min^−1^. Before illumination, the system was stabilized in the dark for 1 h to get a stable baseline. Afterwards, the suspension was illuminated employing an Osram XBO 1000 W Xe-Lamp (Müller) equipped with a 420 nm/500 nm cutoff filter.

### Electron paramagnetic resonance (EPR) spectroscopy

EPR measurements were carried out with a MiniScope MS400 spectrometer (Magnettech GmbH, Germany). A 500 W Mercury-Lamp (Muller) equipped with an optical cutoff filter (GC420/GC500) enables the illumination in the UV-Vis and visible light region. For low temperature measurements (90 K), the samples were placed in X-Band standard EPR tubes (Wilmad, 2 mm, O.D.) and cooled with a commercial EPR cold finger quartz Dewar by applying liquid nitrogen. The *g* values were calculated with the formula *hν* = *gβB*_0_ (*B*_0_ – external magnetic field, *β* – Bohr magneton, *g*_e_ – Landé *g*-factor) as described in the literature.^4*a*^ The frequency was at around 9.42 GHz, and the *B*_0_ was varied between 305–365 mT.

### Laser flash photolysis spectroscopy

During the laser flash photolysis measurements the samples were excited with an Nd-YAG laser (Brilliant B, Quantel) and the spectra were monitored by a laser flash photolysis spectrometer (LKS 80, Applied Photophysics). The excitation wavelength was tuned to 420 nm by employing an Optical Parametric Oscillator (OPO) (MagicPRISM, OPOTEK Inc.). Together with the OPO the 3rd harmonic (355 nm) of the Brilliant B laser was used to generate the visible light pulses with 6 mm diameter and 6 ns pulse length. The 532 nm laser was supplied by the 2nd harmonic (532 nm) of the Brilliant B laser. All experiments were performed in diffuse reflectance mode, where the analyzing light (supplied by a 150 W xenon arc lamp) was focused onto the sample and the reflected light was guided through the monochromator into the detector (Hamamatsu R928 photomultiplier). The light level detected by the oscilloscope was kept at 100 mV for all measurements to compensate the wavelength dependant sensitivity. Furthermore, all examinations were conducted under N_2_ atmosphere and 12 shots were averaged for every transient. The energy density of the 420 nm and 532 nm laser beam was around 5 mJ cm^−2^ per shot, respectively. The transient absorption change in reflectance Δ*J* was calculated from the absorbance values according to the literature,^18^ which also provides more detailed information about the set-up.

### Theoretical calculation details

All spin-polarized calculations of optimization and energy bands were performed by Cambridge Sequential Total Energy Package (CASTEP). Projected augmented wave (PAW) pseudopotential was used to describe core electrons and plane wave basis set with 380 eV to represent valance electrons. Exchange-correlation energy was approximated by Perdew–Burke–Ernzerhof (PBE) functional of the generalized gradient approximation (GGA). Since the majority of P25 is anatase, we chose it for calculation. The model for anatase TiO_2_ dominant (101) surface was represented with a 12 monolayer unit cell with a 15 Å vacuum slab. Au–TiO_2_ model was constructed with a two gold atom cluster, to get rid of multi isomers of Au clusters,^19–21^ and TiO_2_ (101) surface, with bottom six atomic layers fixed and upper six layers relax (ESI Fig. S4†). A 4 × 4 × 2 *k*-points girds was used to optimize anatase cell, 2 × 2 × 1 *k*-points girds was used for TiO_2_ (101) surface and Au–TiO_2_ optimization and energy bands calculation both. The optimized lattice values of anatase were 3.78 and 9.49 Å, close to experimental values (3.78 and 9.5 Å). To overcome the self-interaction error of GGA method, a Hubbard correction (DFT+U) of 8.3 eV was imposed on Ti d-states; a good qualitative description of the electronic was obtained (Fig. S5†) with 3.23 eV band gap for bulk and 3.43 eV for (101) surface. No correction was applied to gold.

## 3. Results

### Material characterization

**Fig. 2:**
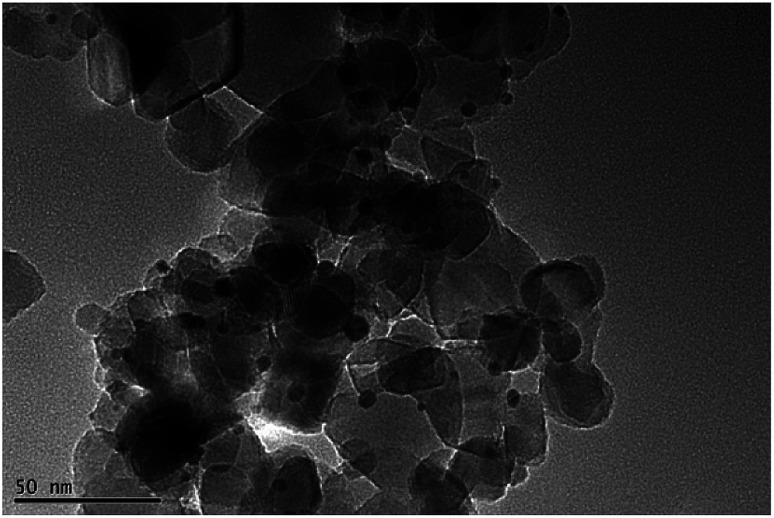
TEM image of Au (1 wt%)–TiO_2_.

Fig. 2 shows the transmission electron microscopy (TEM) image of Au–TiO_2_. As can be seen, the lighter particles are the TiO_2_ matrix and the darker particles are the Au nanoparticles. The TEM images of Au–TiO_2_ reveal that the Au nanoparticles are well deposited on the surface of TiO_2_ matrix exhibiting particle sizes between 5 nm and 10 nm. Besides, the contents of Au–TiO_2_ photocatalyst were analyzed by means of X-Ray Diffraction (XRD). Accordingly, in addition to the peaks of anatase TiO_2_ (JCPDS # 01-070-7347) and rutile TiO_2_ (JCPDS # 03-065-5714), the XRD pattern exhibited peaks for Au (JCPDS # 03-065-2870), as shown in Fig. S1.† Due to the low concentration, the peaks of Au are weak. However, no peaks for other impurities (*e.g.* carbon or PVA) were detected within the detection limit.

**Fig. 3:**
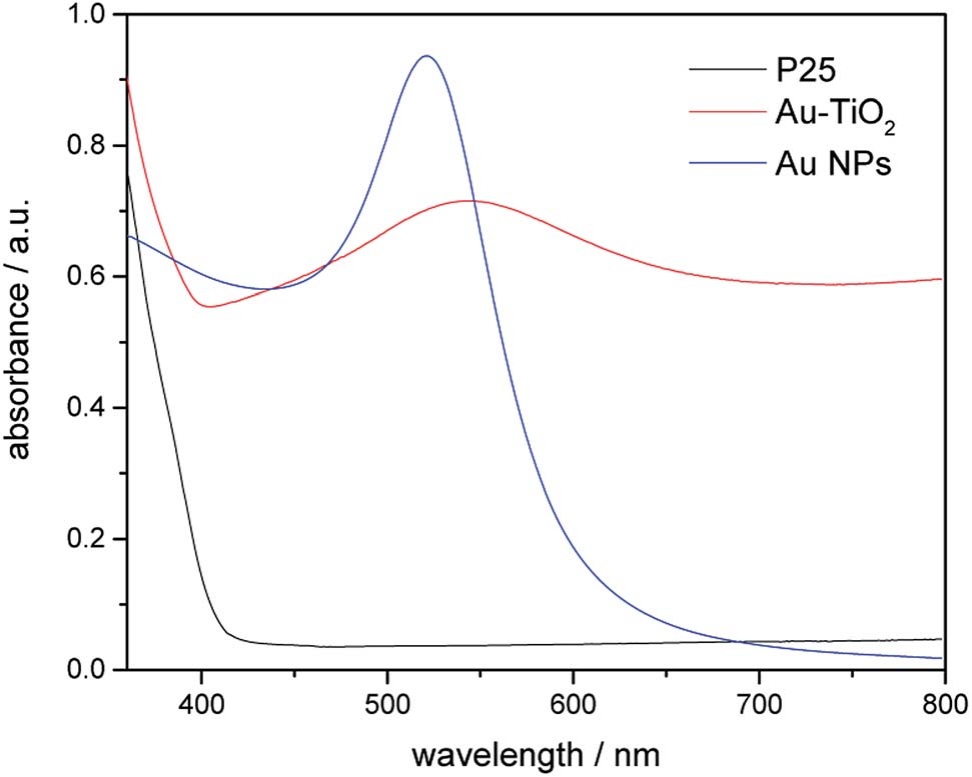
Diffuse-reflectance UV-Vis spectra of bare TiO_2_ (black), Au NPs (blue) and Au–TiO_2_ (red).

In this study, the optical properties of Au nanoparticles, bare TiO_2_ and Au–TiO_2_ were characterized by UV-Vis spectroscopy. As Fig. 3 shows Au-related samples exhibit plasmon band maxima located above 500 nm, namely Au nanoparticles with *λ*_max_ at 520 nm, Au–TiO_2_ with *λ*_max_ at 543 nm, respectively. The absorption edge of bare TiO_2_ is located around 400 nm, which is in good agreement with previous studies.^4*b*^ Spectra of bare TiO_2_ and the employed filters are presented in the ESI Fig. S2.†

### Photocatalytic H_2_ evolution test

**Fig. 4:**
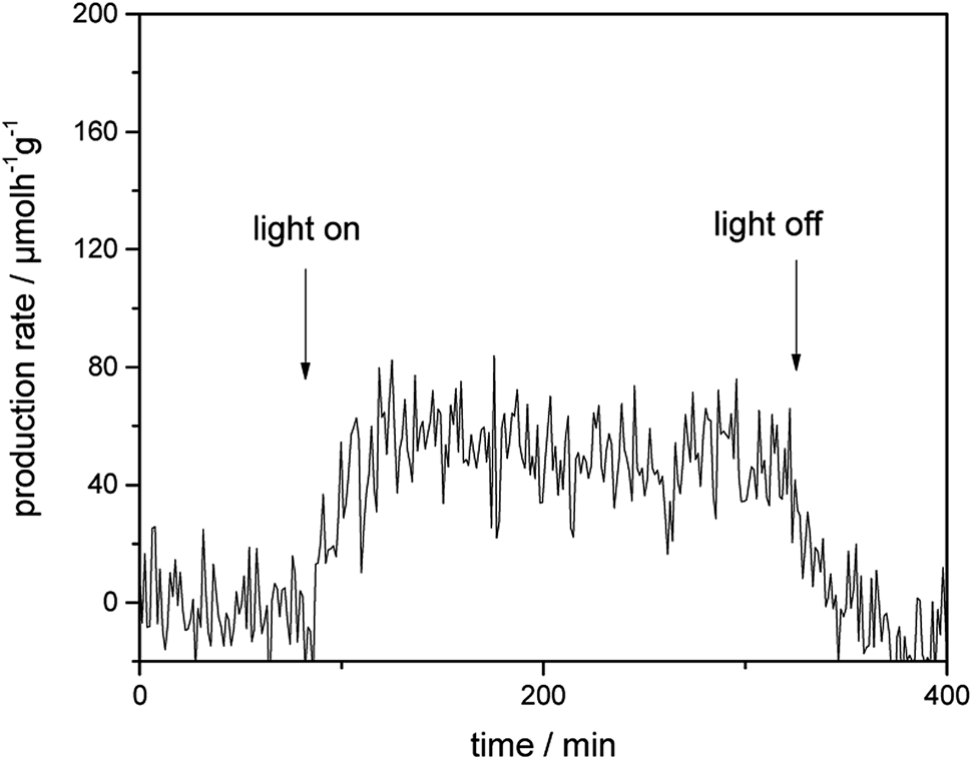
Photocatalytic H_2_ evolution rate of Au–TiO_2_ obtained from CH_3_OH/H_2_O mixtures upon visible light illumination in the presence of a 500 nm cutoff filter.

Fig. 4 presents the photocatalytic H_2_ evolution rates from CH_3_OH/H_2_O mixtures obtained employing Au-loaded TiO_2_ under visible-light illumination in the presence of a 500 nm cutoff filter. However, no formation of H_2_ was detected for bare TiO_2_ photocatalyst within the detection limit under the same reaction condition. In comparison to bare TiO_2_, Au–TiO_2_ exhibited H_2_ production ability upon visible light illumination in the presence of a 500 nm cutoff filter, indicating that Au-SPR can directly lead to photocatalytic H_2_ production.

### EPR analysis

The photoinduced EPR signals of trapped electrons and holes for bare TiO_2_ and Au–TiO_2_ were recorded in Ar at 90 K by (applying liquid nitrogen). The EPR parameters of the detected signals and their assignments were presented (ESI Fig. S3 and Table S1†), which are in good agreement with the previous literatures.^4,22–24^

**Fig. 5:**
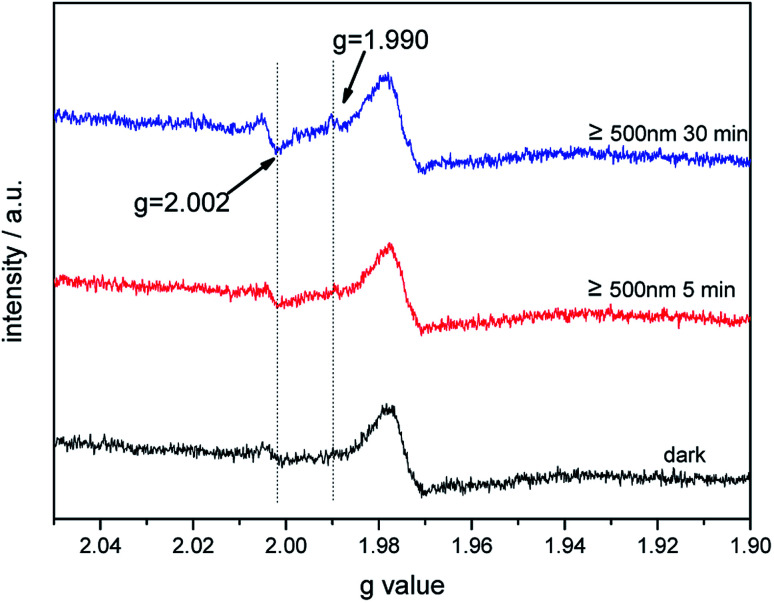
EPR spectra of Au–TiO_2_ obtained in the dark and under visible light illumination (≥500 nm) at 90 K.

In the presence of a 500 nm cutoff filter, when comparing the EPR signals of bare Au–TiO_2_ obtained in the dark with those observed upon visible light illumination (≥500 nm), small but clear signal of trapped electrons (anatase Ti^3+^ at *g* = 1.990) was observed (see Fig. 5), indicating the photo-induced electrons were trapped in TiO_2_.

### Laser flash photolysis analysis

**Fig. 6:**
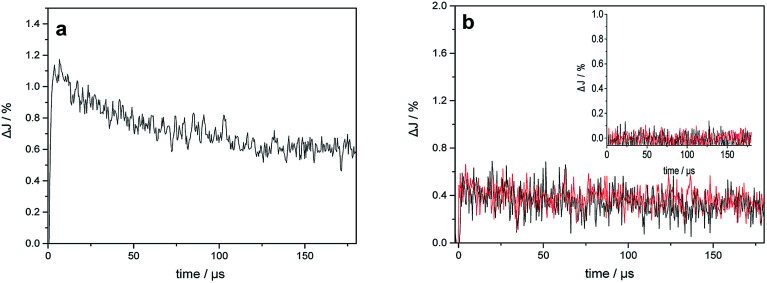
(a) Transient absorption signals of bare TiO_2_ observed at 680 nm upon 420 nm laser excitation; (b) transient absorption signals of bare TiO_2_ (inset) and Au–TiO_2_ observed at 680 nm (black) and 400 nm (red) upon 532 nm laser excitation in a N_2_ atmosphere.

Fig. 6 shows the transient absorption signals measured at 400 nm and 680 nm for bare TiO_2_ and Au–TiO_2_ in the absence of any electron acceptor or donor. From the literature, it is known that Ti^3+^ species absorb in the wavelength range around 680 nm,^25*a*^ while the signal of the trapped holes can be monitored at 400–550 nm.^25*b*^

As shown in Fig. 6a, the transient signal of the trapped electron in TiO_2_ was detected indicating that bare TiO_2_ can be directly excited by 420 nm laser beam. The decay of the transient signals can be related to the internal electron/hole recombination.^18,25^ Upon 532 nm laser excitation, which is the plasmon band of the Au NPs, in addition to the transient absorption signal of the trapped electrons at 680 nm, the signal of the trapped holes in TiO_2_ (see Fig. 6b) was monitored at 400 nm. As expected, no transient signals were observed for bare TiO_2_ in the blank experiment (see Fig. 6b inset). The trapped holes observed at 400 nm exhibit nearly the same transient signal intensity and kinetic like the trapped electrons detected at 680 nm.

### DFT calculation analysis

**Fig. 7:**
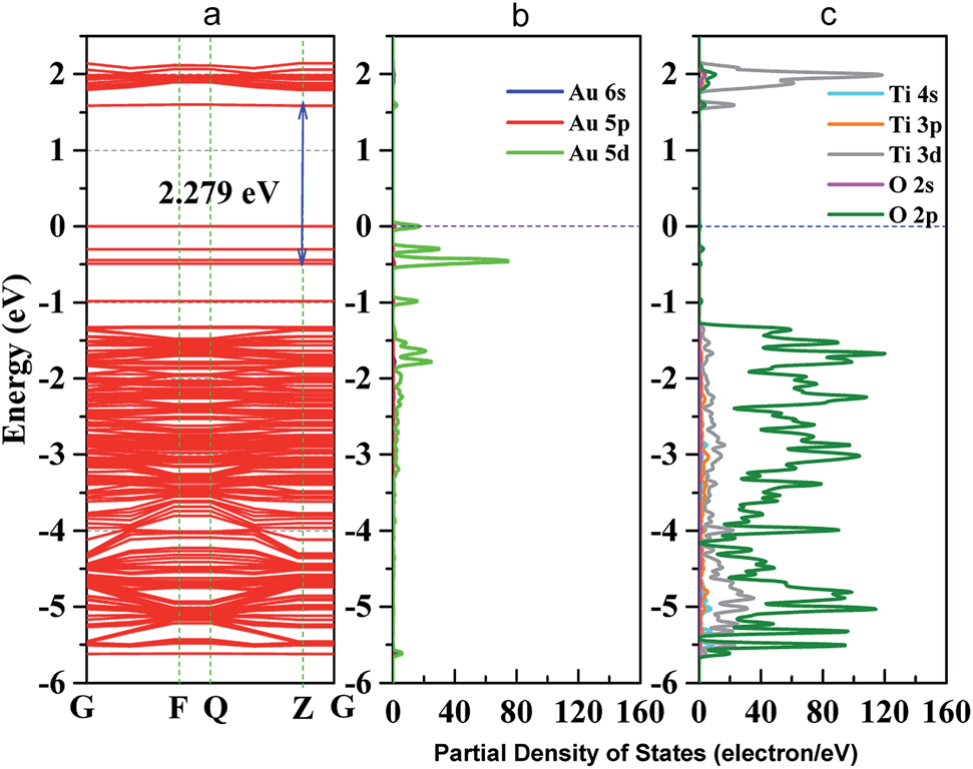
(a) band structure of Au–TiO_2_ slab; (b) partial density of states (PDOS) of Au cluster; (c) PDOS of TiO_2_ (101) surface; blue dot line represents Fermi level.

To further analyze the enhanced photocatalytic activities of Au–TiO_2_, the electronic structure of Au–TiO_2_ system were calculated, showed in Fig. 7. As shown in Fig. 7a, energy levels of the system are apparently split due to the reduction of symmetry and the existence of surface dangling bonds. Moreover, new energy levels are appeared, two near the top of valence band and bottom of conduction band each, and four near Fermi level. Meanwhile, Fig. 7 presented clear evidence that new isolated impurity energy levels are mainly attributed to d orbitals of Au clusters, and the top of valence band by the hybrid orbitals of Au and TiO_2_ surface.

## 4. Discussion

Obviously, Au–TiO_2_ can produce H_2_ upon visible light illumination (employing a 500 nm cutoff filter). However, bare TiO_2_ absorbs no light in the wavelength range above 500 nm and no H_2_ can be formed (≥500 nm) as evidenced by blank experiments. Besides, the XRD pattern showed no other impurities (*e.g.* carbon or PVA) were detected within the detection limit. Hence, it can be excluded that the visible-activity of Au–TiO_2_ above 500 nm is caused by a carbon radical stem from polyvinyl alcohol remaining in the catalyst from the synthesis. The observation of any H_2_ and signals of trapped electrons and holes can only be related to the Au-SPR effect. As shown in Fig. S3,† the appearance of new EPR signals can be observed upon illumination (≥500 nm) which can be attributed to trapped electrons and trapped holes according to the literature.^4,22–24^ The new formed EPR signals indicate the excitation of Au–TiO_2_ photocatalyst upon visible light illumination above 500 nm. However, as new formed EPR signals are indistinctive (especially the signals at *g* = 2.002–2.005 (ref. 4 and 22−24)) laser excitation was employed to totally get rid of the confusion caused by the proximity of *g* values for signals of trapped electrons and trapped holes.

Upon 532 nm laser (∼2.34 eV) excitation, the transient signals of the trapped electrons and holes in TiO_2_ were also observed, which further confirmed the presence of photogenerated electrons and holes. It should be noted here that, the transient signals of the Au-SPR induced electrons can be detected in ps scale^14^ and most charges recombine within 1 ns.^26,27^ Therefore, the significant decay process of the transient signals in Fig. 6b was not observed within the detection time scale. In the absence of any electron acceptor or donor, transient signals of the trapped electrons and holes in TiO_2_ can be observed on longer time scale (μs), as presented. The long-lived charges can be correlated with the photocatalytic activity of the photocatalysts.^28,29^

**Fig. 8:**
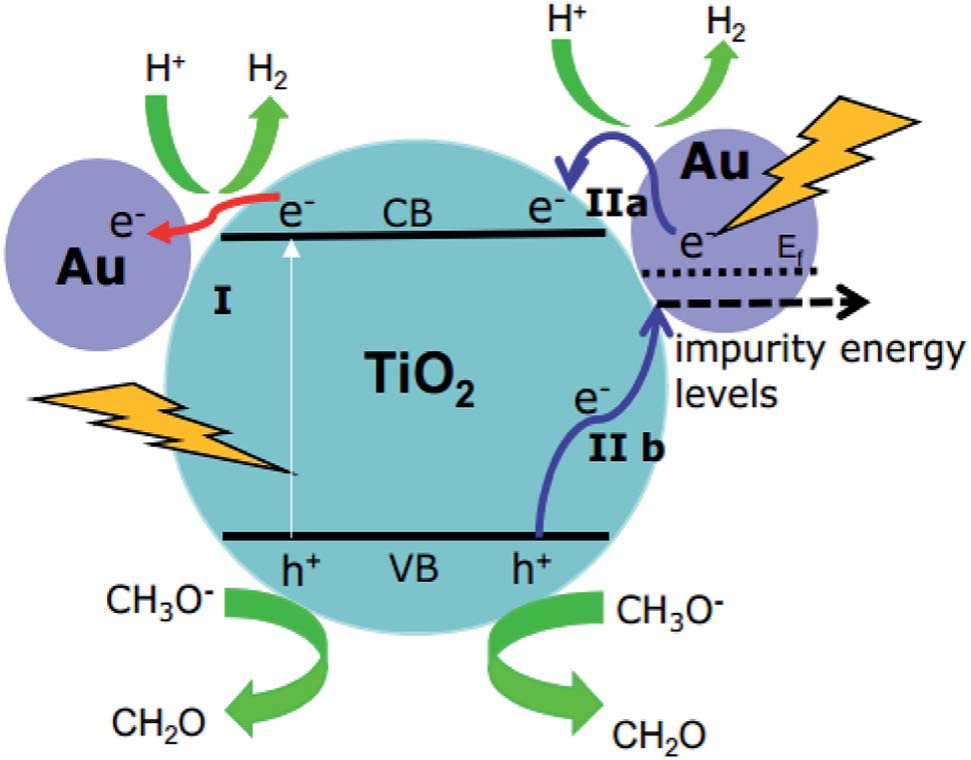
Proposed mechanisms for H_2_ production using Au–TiO_2_ in water/methanol mixtures upon visible light illumination (≥420 nm: process I + II; ≥500 nm: process II).

It should be emphasized here that the energy of the incoming photons (*λ* ≥ 500 nm, *E*_*hν*_ ≤ 2.48 eV) is insufficient to excite the electrons from the valence band to the conduction band of TiO_2_ and leave the holes in the valence band of TiO_2_. However, due to the Au-SPR, many electrons in Au have higher energy than the conduction band of TiO_2_, facilitating the electron injection from Au to the conduction band of TiO_2_.^13,30^ This provided a reasonable explanation for the observation of the Ti^3+^ in both EPR spectroscopy and laser flash photolysis spectroscopy. As reported,^13,30^ the direct electron injection process is thermodynamically favorable, since the photon energy (*hν*) only needs to overcome the gap between the Fermi level (*E*_f_) of Au and the top of TiO_2_ conduction band (*E*_CB_), that is: *hν* ≥ *E*_CB0_ – *E*_f_. Moreover, after the contact of Au and TiO_2_, the Fermi level of the Au nanoparticles will be equalized to the work function and the conduction band of TiO_2_ will be bent down.^30^ Therefore, the trapped electrons leading to the formation of Ti^3+^ are from the “hot electron injection” of the Au nanoparticles, process II (a) in Fig. 8.

On the other hand, since no holes can be transferred from the Au nanoparticles to the valence band of TiO_2_, the EPR signals of the trapped holes in TiO_2_ (signal D) can only originate from the Au-SPR initiated electron–hole pair generation in TiO_2_. However, it should be noted here again that the energy of the visible light (≥500 nm, *i.e.*, ≤2.5 eV) is not sufficient to overcome the band gap energy of TiO_2_. In this case, it is assumed that the Au-SPR initiates the Interfacial Charge Transfer (IFCT), as proposed by Creutz.^31^ Accordingly, electrons in the valence band of TiO_2_ directly transfer to the surface Au clusters, that is not *via* the excited state,^32^ while the holes are trapped in the valence band of TiO_2_, process II (b) in Fig. 8. Besides, after electrons injection from Au to the conduction band of TiO_2_, monovalent of Au might be generated. Therefore, the DET process also provides the possibility of interfacial charge transfer from valence band of TiO_2_ to monovalent of Au.

Moreover, the obtained theoretical calculation results provide further evidences supporting the above proposed mechanisms. Firstly, a good qualitative description of the electronic was obtained (Fig. S5†) with 3.23 eV band gap for bulk and 3.43 eV for (101) surface. As presented above (Fig. 7), with the deposition of Au clusters on the surface of TiO_2_, new isolated impurity energy levels appeared mainly due to d orbitals of Au clusters. Therefore, the possibility of electron transfer from Au to the conduction band of TiO_2_ is demonstrated by DFT calculation. Besides, the existence of Au impurity energy levels in band gap also provide channels for electrons to be excited with lower energy (*λ* ≥ 500 nm, *E*_*hν*_ ≤ 2.48 eV). Moreover, the maxima absorption wavelength of Au–TiO_2_ in Fig. 3 (543 nm, 2.288 eV) is in good accordance with the energy difference between an impurity level and the bottom of conduction band in DFT result (2.279 eV). Furthermore, due to the small energy gaps, the electron transfer from the valence band of TiO_2_ to the surface loaded Au clusters is thermodynamically feasible, as proposed by IFCT process. Herein, the proposed mechanisms based on the experimental data are favored by the DFT calculation results. In case of the electron transfer processes can occur employing pure anatase as TiO_2_ source, the same process should be easier in the case of using the mixture of anatase and rutile TiO_2_, for example, in Evonik Aeroxide P25 as TiO_2_ source.

Besides, the EPR and the laser flash photolysis spectra (ESI Fig. S3† and 6a) show clear evidence that bare TiO_2_ (P25) can be directly excited by visible light illumination (*λ* ≥ 420 nm) or 420 nm laser beam (process I in Fig. 8), which has long been ignored by some studies. Therefore, the investigation of SPR influence on the visible light photocatalytic activity of TiO_2_ employing a 400/420 nm cutoff filter does not seem to be correct. Actually, upon visible light illumination (*λ* ≥ 420 nm), the direct excitation of TiO_2_ and the SPR-induced excitation can both lead to the visible light photocatalytic activity of Au–TiO_2_, process I + II in Fig. 8.

Therefore, upon illumination above 500 nm, due to the Au-SPR, electrons can be directly injected into the conduction band of TiO_2_, as reported previously.^4*a*,10,13,14,26^ Simultaneously, the Au-SPR can also initiate electron hole pair generation in TiO_2_, while the electrons directly transfer from the valence band of TiO_2_ to the surface Au-related species (IFCT process). The two pathways cooperatively contribute to the SPR-induced visible-driven photocatalytic H_2_ production ability of Au–TiO_2_ photocatalyst. This also provides a reasonable explanation for the photocatalytic activity when the plasmonic metal nanoparticles were homogeneously covered with an insulating SiO_2_ shell.

## 5. Conclusions

In conclusion, Au-SPR driven photocatalytic H_2_ production was observed upon visible light illumination (≥500 nm). Direct experimental evidences were presented that Au nanoparticles can transfer the SPR-induced hot electrons into the conduction band of TiO_2_ upon visible light illumination in the wavelength range above 500 nm. Simultaneously, the Au-SPR can also initiate electron–hole pair generation in TiO_2_. However, due to the photons energy/*E*_SPR_ is insufficient to overcome the band gap of TiO_2_, the initiated electrons directly transfer to the surface Au-related species through the IFCT process rather than *via* the excited state. The experimental results are in good agreement with the data obtained from DFT calculation. The DFT calculation analysis clearly shows how the d orbitals of Au clusters create impurity energy levels and decrease the band gap of Au–TiO_2_. We are optimistic that our contribution may contribute to the controversial discussion about the origin of the SPR- induced electron formation within Au–TiO_2_ thus providing a new horizon for further investigations on exploring more effective visible light harvesting photocatalysts for solar energy conversion.

## Conflicts of interest

There are no conflicts to declare.

## Supplementary Material

RA-008-C8RA05450A-s001
