# Supplementary material for: New insights into the surface plasmon resonance (SPR) driven photocatalytic H2 production of Au–TiO2
Source: RSC Adv. 2018 Jul 19;8(46):25881–7. doi: 10.1039/c8ra05450a (PMC9082917; doi:10.1039/c8ra05450a)
Supplement: RA-008-C8RA05450A-s001 [file RA-008-C8RA05450A-s001.pdf]

Electronic Supplementary Material (ESI) for RSC Advances.
This journal is © The Royal Society of Chemistry 2018

New Insights into the Surface Plasmon Resonance (SPR) Driven Photocatalytic H_2 Production of Au-TiO_2

Jinlin Nie[1,2], Jenny Schneider[2], Fabian Sieland[2], Long Zhou[1], Shuwei Xia[1]*, and Detlef W. Bahnemann[2,3]

(1) Key Laboratory of Marine Chemistry Theory and Technology, Ministry of Education, College of Chemistry and Chemical Engineering, Ocean University of China, Songling Road 238, 266100, Qingdao China

(2) Institut für Technische Chemie, Leibniz Universität Hannover, Callinstr.3, D-30167 Hannover, Germany.

(3) Laboratory "Photoactive Nanocomposite Materials", Saint-Petersburg State University, Ulyanovskaya str. 1, Peterhof, Saint-Petersburg, 198504 Russia

Corresponding Authors: shuweixia@ouc.edu.cn; bahnemann@iftc.uni-hannover.de

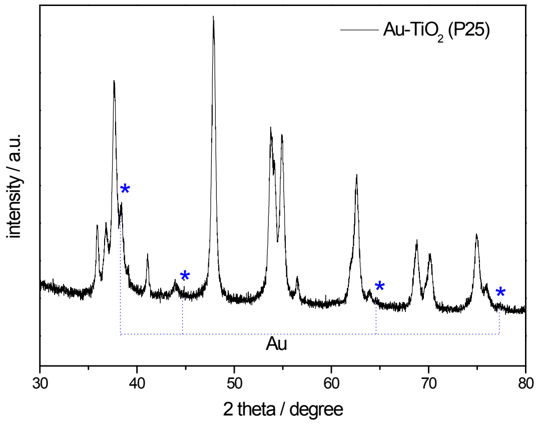

Figure S1. XRD patter of Au-TiO_2 (P25)

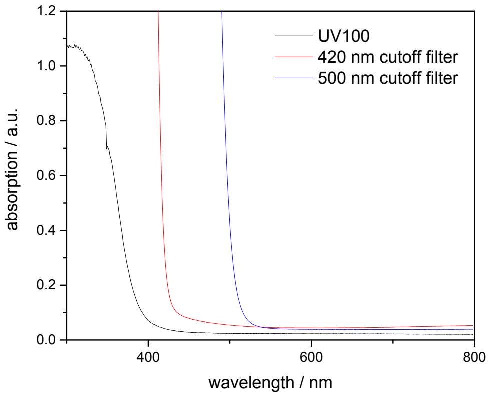

Figure S2: The UV-Vis spectra of bare TiO_2 and of the employed 420 nm (red) and 500 nm cutoff filters (blue).

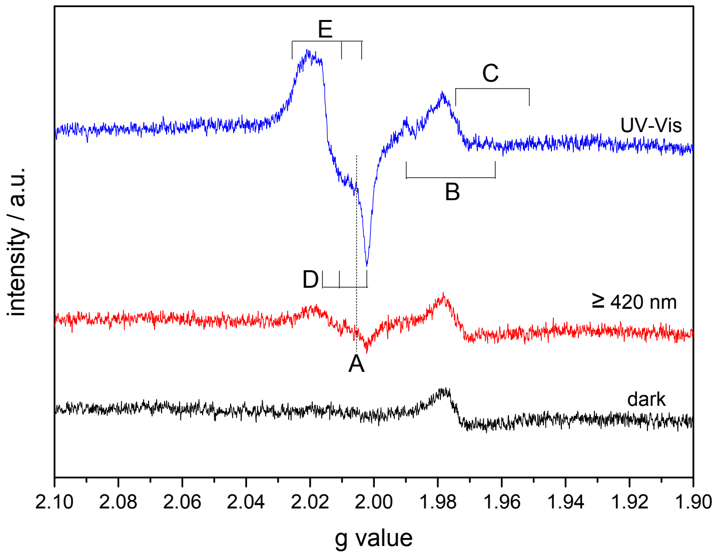

Figure S3. EPR spectra of bare TiO_2 obtained in the dark (black), under visible light illumination (≥ 420 nm) (red) and under UV-Vis (blue) light illumination at 90 K

As shown in Figure S3, upon UV-Vis light illumination electrons trapped at O vacancies (signal A) [4a] and at oxygen molecules (signal E) [22,23] were detected, as well as electrons trapped at Ti^{4+} centers forming the anatase Ti^{3+} (signal C) and rutile Ti^{3+} (signal D) species [4,24]. The signal of trapped holes at O^{2-} forming paramagnetic $O^{\bullet -}$ species (signal D) [23] was also observed. Besides, it should be noted here that signals of trapped electrons and holes were detected for bare TiO_2 upon visible light illumination (≥ 420 nm), indicating that bare TiO_2 can be excited by visible light illumination at around 420 nm. The detected signals and their respective assignments are summarized in Table S1.

Table S1: EPR parameters of detected signals shown in Figure 3 and their assignments based on the literature data.

**Signal**	**assignment**		**g value**	
--	--	g_1	g_2	g_3
**A**	e^- trapped at O vacancies	2.005	2.005	2.005 [4]
**B**	anatase Ti^{3+}	1.990	1.990	1.957 [22]
**C**	rutile Ti^{3+}	1.975	1.975	1.951 [4]
**D**	Ti^{4+}-$O^{\bullet -}$ -Ti^{4+}-OH^-	2.016	2.012	2.002 [23]
**E**	Ti^{4+}-$O_2^{\bullet -}$	2.026	2.010	2.003 [4]

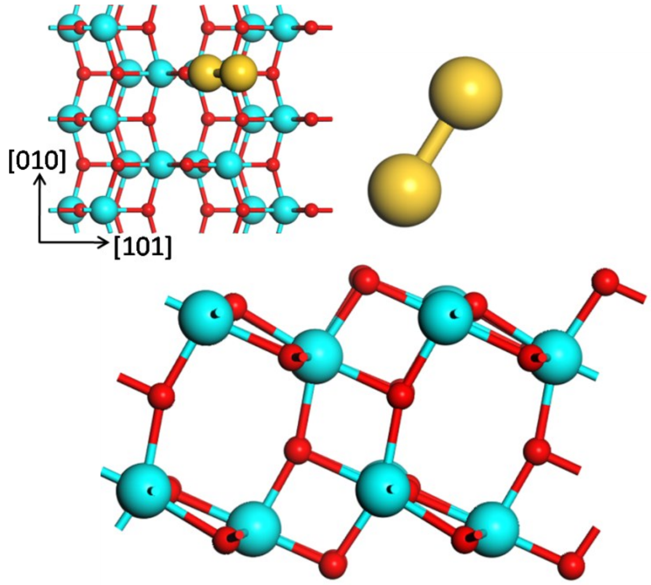

Figure S4: Side and top view of Au-TiO_2 model, red, blue and golden balls are O, Ti and Au atom, respectively.

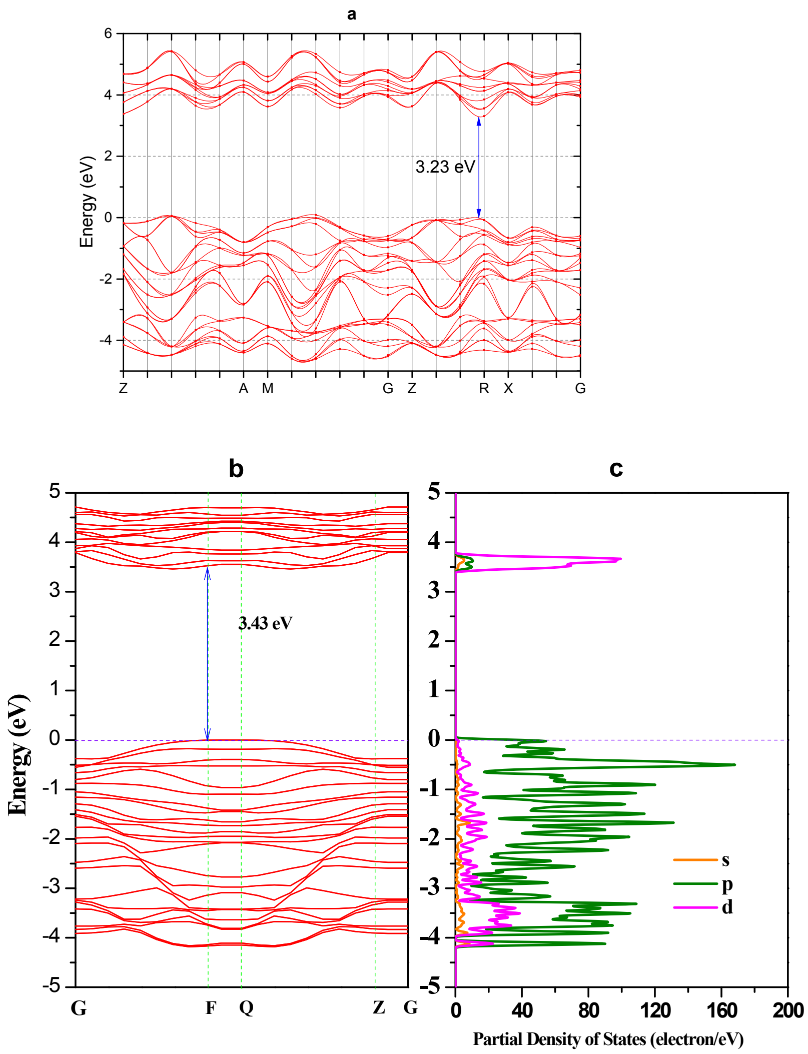

Fig. S5: a) band structure of bulk anatase, b) (101) Surface and c) partial density of states of TiO_2 (101) surface
